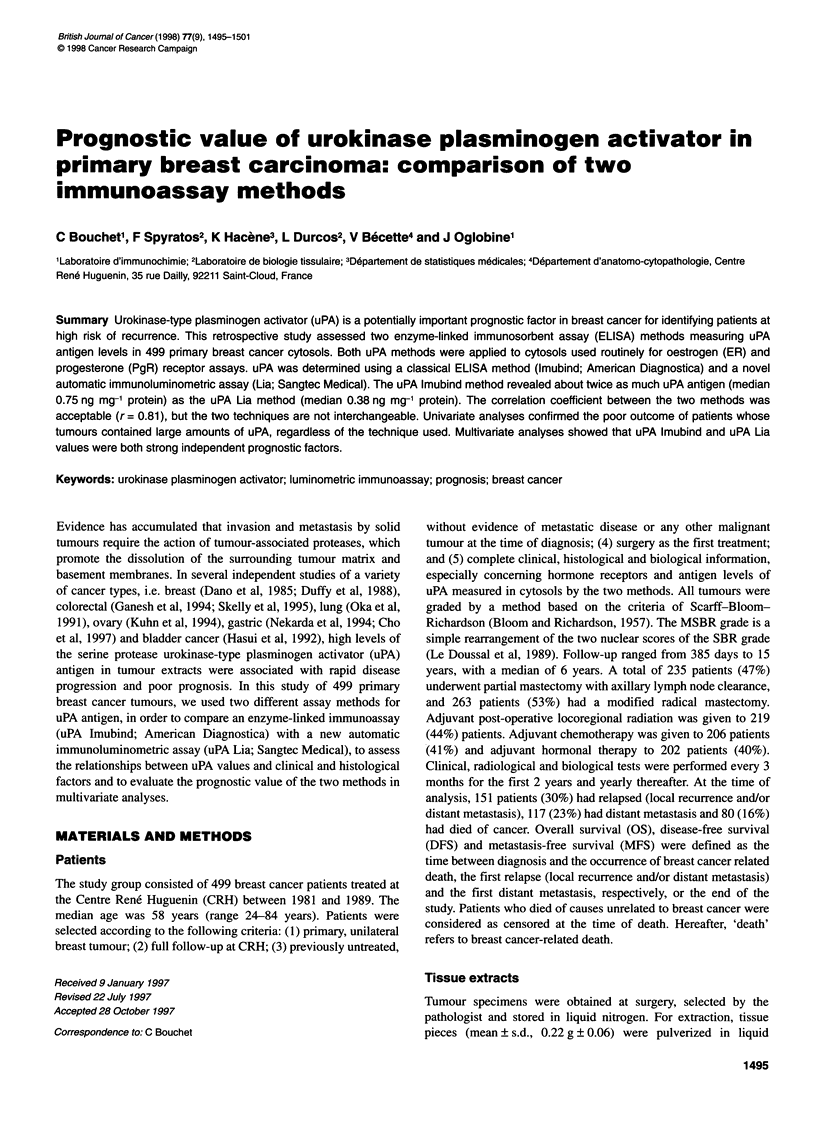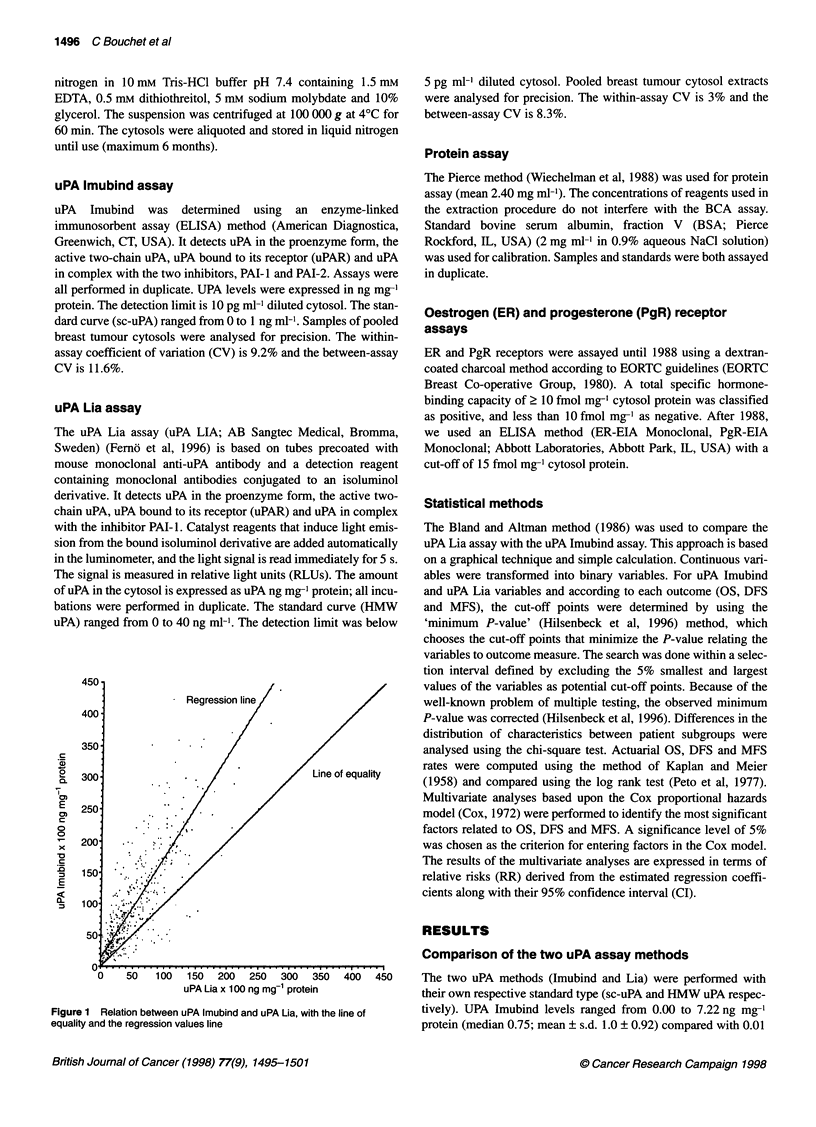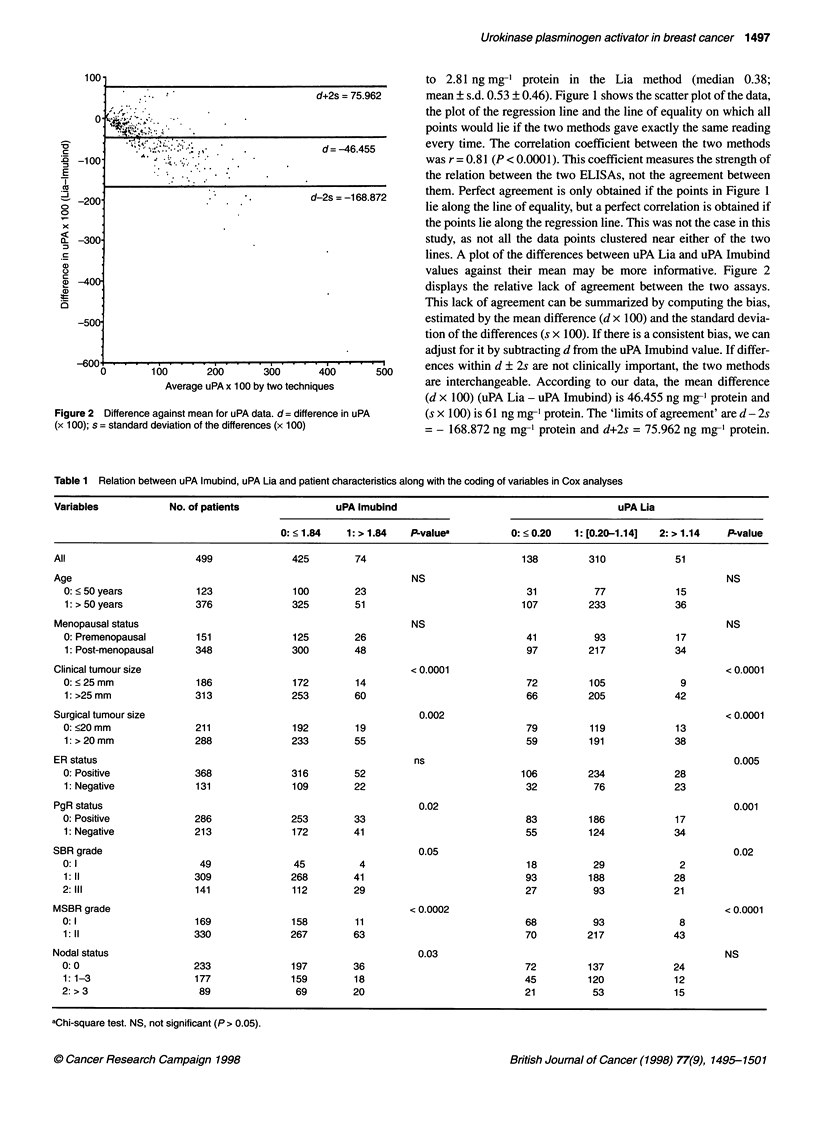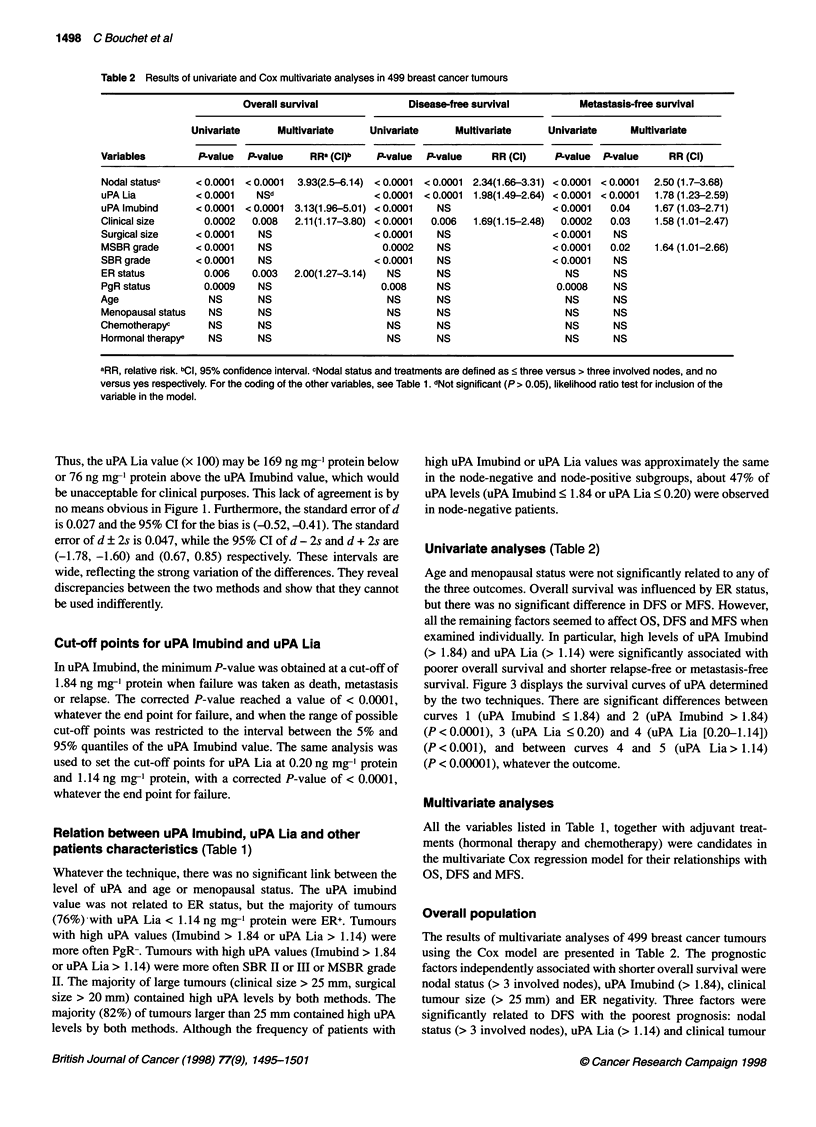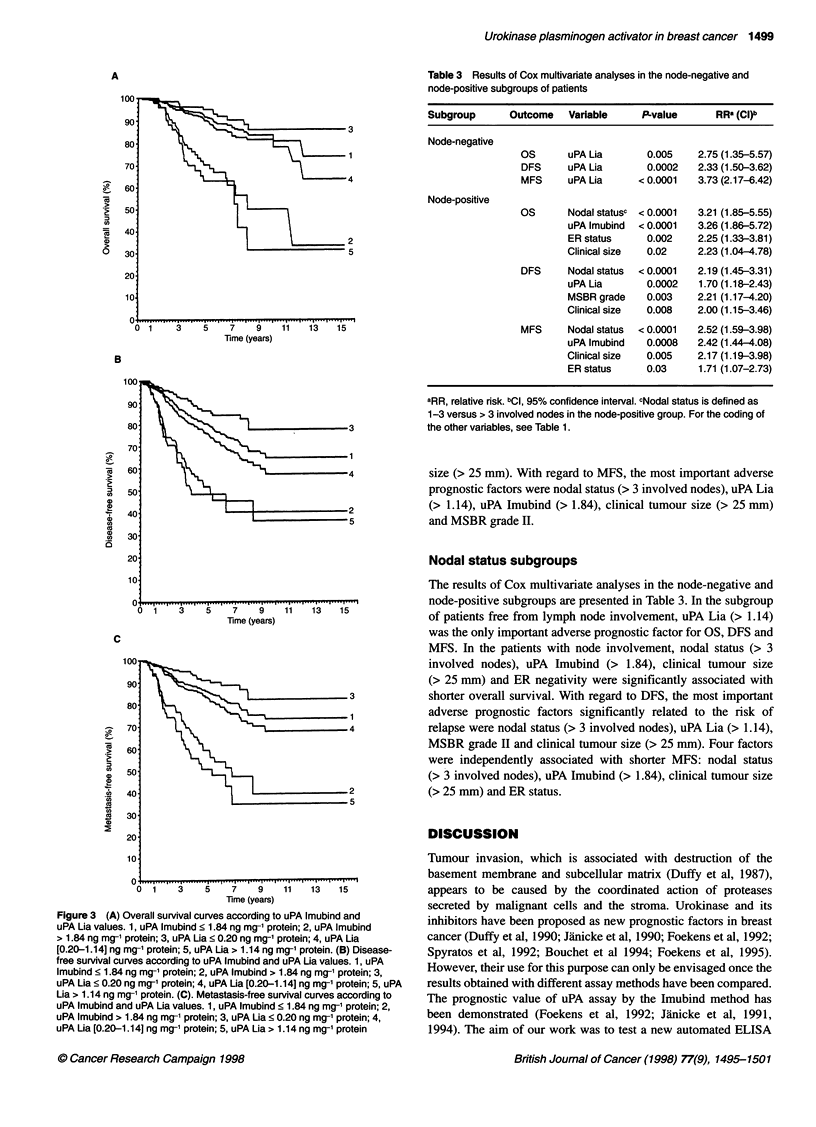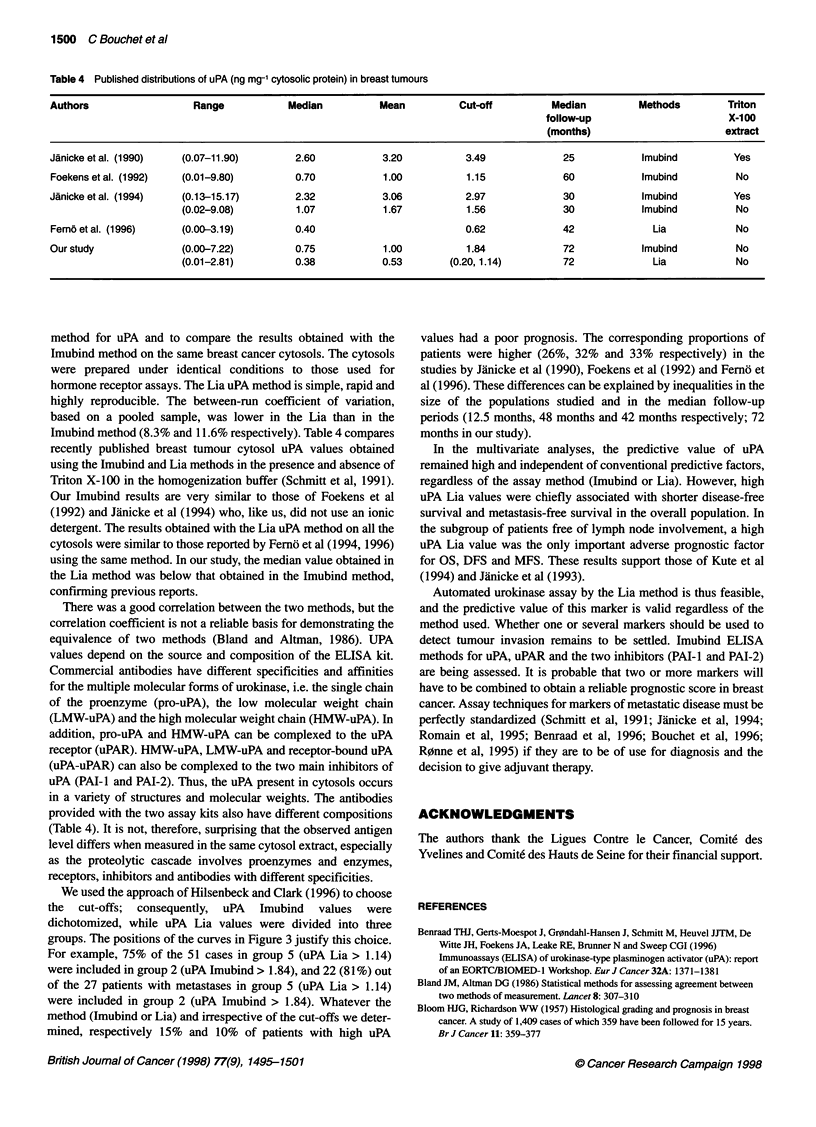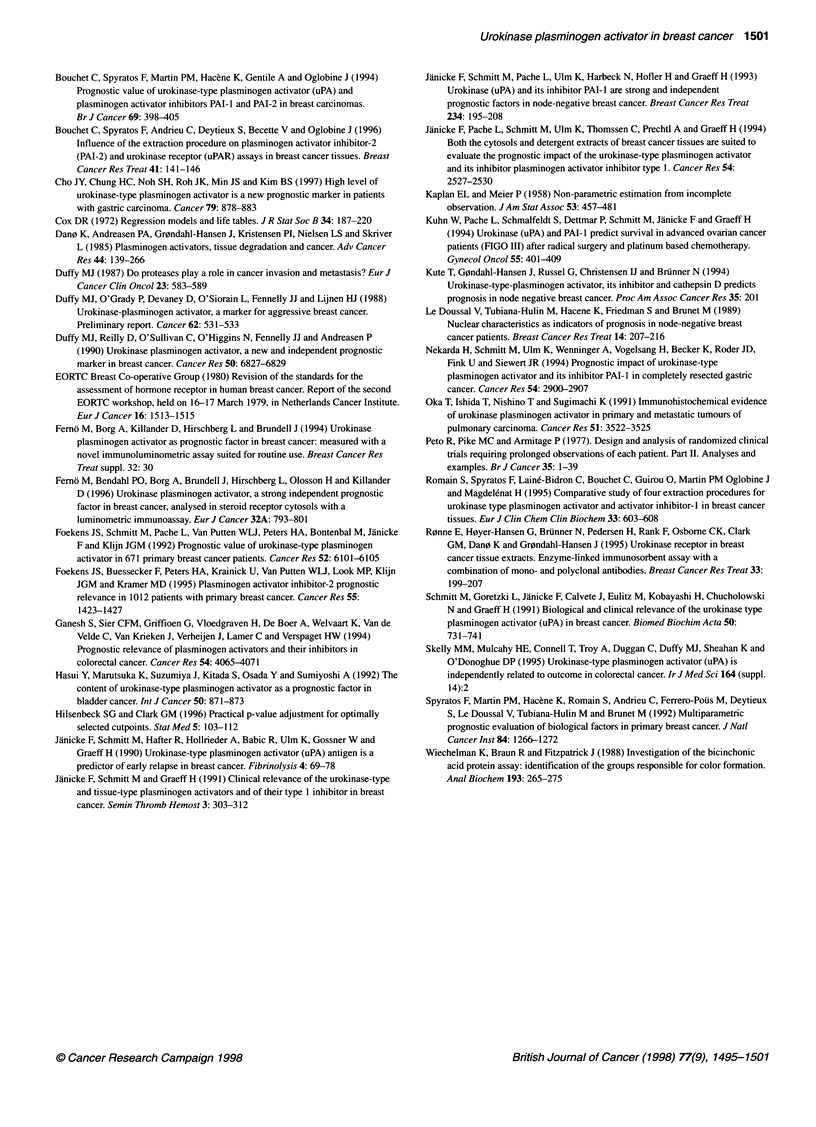# Prognostic value of urokinase plasminogen activator in primary breast carcinoma: comparison of two immunoassay methods.

**DOI:** 10.1038/bjc.1998.246

**Published:** 1998-05

**Authors:** C. Bouchet, F. Spyratos, K. HacÃ¨ne, L. Durcos, V. BÃ©cette, J. Oglobine

**Affiliations:** Laboratoire d'immunochimie, Centre RenÃ© Huguenin, Saint-Cloud, France.

## Abstract

Urokinase-type plasminogen activator (uPA) is a potentially important prognostic factor in breast cancer for identifying patients at high risk of recurrence. This retrospective study assessed two enzyme-linked immunosorbent assay (ELISA) methods measuring uPA antigen levels in 499 primary breast cancer cytosols. Both uPA methods were applied to cytosols used routinely for oestrogen (ER) and progesterone (PgR) receptor assays. uPA was determined using a classical ELISA method (Imubind; American Diagnostica) and a novel automatic immunoluminometric assay (Lia; Sangtec Medical). The uPA Imubind method revealed about twice as much uPA antigen (median 0.75 ng mg(-1) protein) as the uPA Lia method (median 0.38 ng mg(-1) protein). The correlation coefficient between the two methods was acceptable (r = 0.81), but the two techniques are not interchangeable. Univariate analyses confirmed the poor outcome of patients whose tumours contained large amounts of uPA, regardless of the technique used. Multivariate analyses showed that uPA Imubind and uPA Lia values were both strong independent prognostic factors.


British Journal of Cancer (1998) 77(9), 1495–1501
© 1998 Cancer Research Campaign

### Prognostic value of urokinase plasminogen activator in primary breast carcinoma: comparison of two immunoassay methods

**C Bouchet[1], F Spyratos[2], K Hacène[3], L Durcos[2], V Bécette[4] and J Oglobine[1]**

[1]Laboratoire d'immunochimie; [2]Laboratoire de biologie tissulaire; [3]Département de statistiques médicales; [4]Département d'anatomo-cytopathologie, Centre René Huguenin, 35 rue Dailly, 92211 Saint-Cloud, France

**Summary** Urokinase-type plasminogen activator (uPA) is a potentially important prognostic factor in breast cancer for identifying patients at high risk of recurrence. This retrospective study assessed two enzyme-linked immunosorbent assay (ELISA) methods measuring uPA antigen levels in 499 primary breast cancer cytosols. Both uPA methods were applied to cytosols used routinely for oestrogen (ER) and progesterone (PgR) receptor assays. uPA was determined using a classical ELISA method (Imubind; American Diagnostica) and a novel automatic immunoluminometric assay (Lia; Sangtec Medical). The uPA Imubind method revealed about twice as much uPA antigen (median 0.75 ng $mg^{-1}$ protein) as the uPA Lia method (median 0.38 ng $mg^{-1}$ protein). The correlation coefficient between the two methods was acceptable ($r = 0.81$), but the two techniques are not interchangeable. Univariate analyses confirmed the poor outcome of patients whose tumours contained large amounts of uPA, regardless of the technique used. Multivariate analyses showed that uPA Imubind and uPA Lia values were both strong independent prognostic factors.

**Keywords:** urokinase plasminogen activator; luminometric immunoassay; prognosis; breast cancer

Evidence has accumulated that invasion and metastasis by solid tumours require the action of tumour-associated proteases, which promote the dissolution of the surrounding tumour matrix and basement membranes. In several independent studies of a variety of cancer types, i.e. breast (Dano et al, 1985; Duffy et al, 1988), colorectal (Ganesh et al, 1994; Skelly et al, 1995), lung (Oka et al, 1991), ovary (Kuhn et al, 1994), gastric (Nekarda et al, 1994; Cho et al, 1997) and bladder cancer (Hasui et al, 1992), high levels of the serine protease urokinase-type plasminogen activator (uPA) antigen in tumour extracts were associated with rapid disease progression and poor prognosis. In this study of 499 primary breast cancer tumours, we used two different assay methods for uPA antigen, in order to compare an enzyme-linked immunoassay (uPA Imubind; American Diagnostica) with a new automatic immunoluminometric assay (uPA Lia; Sangtec Medical), to assess the relationships between uPA values and clinical and histological factors and to evaluate the prognostic value of the two methods in multivariate analyses.

### MATERIALS AND METHODS

### Patients

The study group consisted of 499 breast cancer patients treated at the Centre René Huguenin (CRH) between 1981 and 1989. The median age was 58 years (range 24–84 years). Patients were selected according to the following criteria: (1) primary, unilateral breast tumour; (2) full follow-up at CRH; (3) previously untreated, without evidence of metastatic disease or any other malignant tumour at the time of diagnosis; (4) surgery as the first treatment; and (5) complete clinical, histological and biological information, especially concerning hormone receptors and antigen levels of uPA measured in cytosols by the two methods. All tumours were graded by a method based on the criteria of Scarff–Bloom–Richardson (Bloom and Richardson, 1957). The MSBR grade is a simple rearrangement of the two nuclear scores of the SBR grade (Le Doussal et al, 1989). Follow-up ranged from 385 days to 15 years, with a median of 6 years. A total of 235 patients (47%) underwent partial mastectomy with axillary lymph node clearance, and 263 patients (53%) had a modified radical mastectomy. Adjuvant post-operative locoregional radiation was given to 219 (44%) patients. Adjuvant chemotherapy was given to 206 patients (41%) and adjuvant hormonal therapy to 202 patients (40%). Clinical, radiological and biological tests were performed every 3 months for the first 2 years and yearly thereafter. At the time of analysis, 151 patients (30%) had relapsed (local recurrence and/or distant metastasis), 117 (23%) had distant metastasis and 80 (16%) had died of cancer. Overall survival (OS), disease-free survival (DFS) and metastasis-free survival (MFS) were defined as the time between diagnosis and the occurrence of breast cancer related death, the first relapse (local recurrence and/or distant metastasis) and the first distant metastasis, respectively, or the end of the study. Patients who died of causes unrelated to breast cancer were considered as censored at the time of death. Hereafter, 'death' refers to breast cancer-related death.

*Received 9 January 1997*
*Revised 22 July 1997*
*Accepted 28 October 1997*

*Correspondence to:* C Bouchet

### Tissue extracts

Tumour specimens were obtained at surgery, selected by the pathologist and stored in liquid nitrogen. For extraction, tissue pieces (mean ± s.d., 0.22 g ± 0.06) were pulverized in liquid nitrogen in 10 mM Tris-HCl buffer pH 7.4 containing 1.5 mM EDTA, 0.5 mM dithiothreitol, 5 mM sodium molybdate and 10% glycerol. The suspension was centrifuged at 100 000 *g* at 4°C for 60 min. The cytosols were aliquoted and stored in liquid nitrogen until use (maximum 6 months).

### uPA Imubind assay uPA Imubind was determined using an enzyme-linked immunosorbent assay (ELISA) method (American Diagnostica, Greenwich, CT, USA). It detects uPA in the proenzyme form, the active two-chain uPA, uPA bound to its receptor (uPAR) and uPA in complex with the two inhibitors, PAI-1 and PAI-2. Assays were all performed in duplicate. UPA levels were expressed in ng $mg^{-1}$ protein. The detection limit is 10 pg $ml^{-1}$ diluted cytosol. The standard curve (sc-uPA) ranged from 0 to 1 ng $ml^{-1}$. Samples of pooled breast tumour cytosols were analysed for precision. The within-assay coefficient of variation (CV) is 9.2% and the between-assay CV is 11.6%.

### uPA Lia assay

The uPA Lia assay (uPA LIA; AB Sangtec Medical, Bromma, Sweden) (Fernö et al, 1996) is based on tubes precoated with mouse monoclonal anti-uPA antibody and a detection reagent containing monoclonal antibodies conjugated to an isoluminol derivative. It detects uPA in the proenzyme form, the active two-chain uPA, uPA bound to its receptor (uPAR) and uPA in complex with the inhibitor PAI-1. Catalyst reagents that induce light emission from the bound isoluminol derivative are added automatically in the luminometer, and the light signal is read immediately for 5 s. The signal is measured in relative light units (RLUs). The amount of uPA in the cytosol is expressed as uPA ng $mg^{-1}$ protein; all incubations were performed in duplicate. The standard curve (HMW uPA) ranged from 0 to 40 ng $ml^{-1}$. The detection limit was below 5 pg $ml^{-1}$ diluted cytosol. Pooled breast tumour cytosol extracts were analysed for precision. The within-assay CV is 3% and the between-assay CV is 8.3%.

**Figure 1** Relation between uPA Imubind and uPA Lia, with the line of equality and the regression values line

### Protein assay

The Pierce method (Wiechelman et al, 1988) was used for protein assay (mean 2.40 mg $ml^{-1}$). The concentrations of reagents used in the extraction procedure do not interfere with the BCA assay. Standard bovine serum albumin, fraction V (BSA; Pierce Rockford, IL, USA) (2 mg $ml^{-1}$ in 0.9% aqueous NaCl solution) was used for calibration. Samples and standards were both assayed in duplicate.

### Oestrogen (ER) and progesterone (PgR) receptor assays

ER and PgR receptors were assayed until 1988 using a dextran-coated charcoal method according to EORTC guidelines (EORTC Breast Co-operative Group, 1980). A total specific hormone-binding capacity of ≥ 10 fmol $mg^{-1}$ cytosol protein was classified as positive, and less than 10 fmol $mg^{-1}$ as negative. After 1988, we used an ELISA method (ER-EIA Monoclonal, PgR-EIA Monoclonal; Abbott Laboratories, Abbott Park, IL, USA) with a cut-off of 15 fmol $mg^{-1}$ cytosol protein.

### Statistical methods

The Bland and Altman method (1986) was used to compare the uPA Lia assay with the uPA Imubind assay. This approach is based on a graphical technique and simple calculation. Continuous variables were transformed into binary variables. For uPA Imubind and uPA Lia variables and according to each outcome (OS, DFS and MFS), the cut-off points were determined by using the 'minimum *P*-value' (Hilsenbeck et al, 1996) method, which chooses the cut-off points that minimize the *P*-value relating the variables to outcome measure. The search was done within a selection interval defined by excluding the 5% smallest and largest values of the variables as potential cut-off points. Because of the well-known problem of multiple testing, the observed minimum *P*-value was corrected (Hilsenbeck et al, 1996). Differences in the distribution of characteristics between patient subgroups were analysed using the chi-square test. Actuarial OS, DFS and MFS rates were computed using the method of Kaplan and Meier (1958) and compared using the log rank test (Peto et al, 1977). Multivariate analyses based upon the Cox proportional hazards model (Cox, 1972) were performed to identify the most significant factors related to OS, DFS and MFS. A significance level of 5% was chosen as the criterion for entering factors in the Cox model. The results of the multivariate analyses are expressed in terms of relative risks (RR) derived from the estimated regression coefficients along with their 95% confidence interval (CI).

### RESULTS

### Comparison of the two uPA assay methods

The two uPA methods (Imubind and Lia) were performed with their own respective standard type (sc-uPA and HMW uPA respectively). UPA Imubind levels ranged from 0.00 to 7.22 ng $mg^{-1}$ protein (median 0.75; mean ± s.d. 1.0 ± 0.92) compared with 0.01

© Cancer Research Campaign 1998

**Figure 2** Difference against mean for uPA data. *d* = difference in uPA (× 100); *s* = standard deviation of the differences (× 100)

to 2.81 ng $mg^{-1}$ protein in the Lia method (median 0.38; mean ± s.d. 0.53 ± 0.46). Figure 1 shows the scatter plot of the data, the plot of the regression line and the line of equality on which all points would lie if the two methods gave exactly the same reading every time. The correlation coefficient between the two methods was $r = 0.81$ ($P < 0.0001$). This coefficient measures the strength of the relation between the two ELISAs, not the agreement between them. Perfect agreement is only obtained if the points in Figure 1 lie along the line of equality, but a perfect correlation is obtained if the points lie along the regression line. This was not the case in this study, as not all the data points clustered near either of the two lines. A plot of the differences between uPA Lia and uPA Imubind values against their mean may be more informative. Figure 2 displays the relative lack of agreement between the two assays. This lack of agreement can be summarized by computing the bias, estimated by the mean difference ($d \times 100$) and the standard deviation of the differences ($s \times 100$). If there is a consistent bias, we can adjust for it by subtracting $d$ from the uPA Imubind value. If differences within $d \pm 2s$ are not clinically important, the two methods are interchangeable. According to our data, the mean difference ($d \times 100$) (uPA Lia – uPA Imubind) is 46.455 ng $mg^{-1}$ protein and ($s \times 100$) is 61 ng $mg^{-1}$ protein. The 'limits of agreement' are $d - 2s$ = – 168.872 ng $mg^{-1}$ protein and $d+2s$ = 75.962 ng $mg^{-1}$ protein.

**Table 1** Relation between uPA Imubind, uPA Lia and patient characteristics along with the coding of variables in Cox analyses

| Variables | No. of patients | uPA Imubind | | | uPA Lia | | | |
|---|---|---|---|---|---|---|---|---|
| | | 0: ≤ 1.84 | 1: > 1.84 | *P*-value[a] | 0: ≤ 0.20 | 1: [0.20–1.14] | 2: > 1.14 | *P*-value |
| All | 499 | 425 | 74 | | 138 | 310 | 51 | |
| Age | | | | NS | | | | NS |
| 0: ≤ 50 years | 123 | 100 | 23 | | 31 | 77 | 15 | |
| 1: > 50 years | 376 | 325 | 51 | | 107 | 233 | 36 | |
| Menopausal status | | | | NS | | | | NS |
| 0: Premenopausal | 151 | 125 | 26 | | 41 | 93 | 17 | |
| 1: Post-menopausal | 348 | 300 | 48 | | 97 | 217 | 34 | |
| Clinical tumour size | | | | < 0.0001 | | | | < 0.0001 |
| 0: ≤ 25 mm | 186 | 172 | 14 | | 72 | 105 | 9 | |
| 1: >25 mm | 313 | 253 | 60 | | 66 | 205 | 42 | |
| Surgical tumour size | | | | 0.002 | | | | < 0.0001 |
| 0: ≤20 mm | 211 | 192 | 19 | | 79 | 119 | 13 | |
| 1: > 20 mm | 288 | 233 | 55 | | 59 | 191 | 38 | |
| ER status | | | | ns | | | | 0.005 |
| 0: Positive | 368 | 316 | 52 | | 106 | 234 | 28 | |
| 1: Negative | 131 | 109 | 22 | | 32 | 76 | 23 | |
| PgR status | | | | 0.02 | | | | 0.001 |
| 0: Positive | 286 | 253 | 33 | | 83 | 186 | 17 | |
| 1: Negative | 213 | 172 | 41 | | 55 | 124 | 34 | |
| SBR grade | | | | 0.05 | | | | 0.02 |
| 0: I | 49 | 45 | 4 | | 18 | 29 | 2 | |
| 1: II | 309 | 268 | 41 | | 93 | 188 | 28 | |
| 2: III | 141 | 112 | 29 | | 27 | 93 | 21 | |
| MSBR grade | | | | < 0.0002 | | | | < 0.0001 |
| 0: I | 169 | 158 | 11 | | 68 | 93 | 8 | |
| 1: II | 330 | 267 | 63 | | 70 | 217 | 43 | |
| Nodal status | | | | 0.03 | | | | NS |
| 0: 0 | 233 | 197 | 36 | | 72 | 137 | 24 | |
| 1: 1–3 | 177 | 159 | 18 | | 45 | 120 | 12 | |
| 2: > 3 | 89 | 69 | 20 | | 21 | 53 | 15 | |

[a]Chi-square test. NS, not significant ($P > 0.05$).

© Cancer Research Campaign 1998

Table 2 Results of univariate and Cox multivariate analyses in 499 breast cancer tumours

| Variables | Overall survival | | | Disease-free survival | | | Metastasis-free survival | | |
|---|---|---|---|---|---|---|---|---|---|
| | Univariate | Multivariate | | Univariate | Multivariate | | Univariate | Multivariate | |
| | P-value | P-value | RR[a] (CI)[b] | P-value | P-value | RR (CI) | P-value | P-value | RR (CI) |
| Nodal status[c] | < 0.0001 | < 0.0001 | 3.93(2.5–6.14) | < 0.0001 | < 0.0001 | 2.34(1.66–3.31) | < 0.0001 | < 0.0001 | 2.50 (1.7–3.68) |
| uPA Lia | < 0.0001 | NS[d] | | < 0.0001 | < 0.0001 | 1.98(1.49–2.64) | < 0.0001 | < 0.0001 | 1.78 (1.23–2.59) |
| uPA Imubind | < 0.0001 | < 0.0001 | 3.13(1.96–5.01) | < 0.0001 | NS | | < 0.0001 | 0.04 | 1.67 (1.03–2.71) |
| Clinical size | 0.0002 | 0.008 | 2.11(1.17–3.80) | < 0.0001 | 0.006 | 1.69(1.15–2.48) | 0.0002 | 0.03 | 1.58 (1.01–2.47) |
| Surgical size | < 0.0001 | NS | | < 0.0001 | NS | | < 0.0001 | NS | |
| MSBR grade | < 0.0001 | NS | | 0.0002 | NS | | < 0.0001 | 0.02 | 1.64 (1.01–2.66) |
| SBR grade | < 0.0001 | NS | | < 0.0001 | NS | | < 0.0001 | NS | |
| ER status | 0.006 | 0.003 | 2.00(1.27–3.14) | NS | NS | | NS | NS | |
| PgR status | 0.0009 | NS | | 0.008 | NS | | 0.0008 | NS | |
| Age | NS | NS | | NS | NS | | NS | NS | |
| Menopausal status | NS | NS | | NS | NS | | NS | NS | |
| Chemotherapy[c] | NS | NS | | NS | NS | | NS | NS | |
| Hormonal therapy[e] | NS | NS | | NS | NS | | NS | NS | |

[a]RR, relative risk. [b]CI, 95% confidence interval. [c]Nodal status and treatments are defined as ≤ three versus > three involved nodes, and no versus yes respectively. For the coding of the other variables, see Table 1. [d]Not significant ($P > 0.05$), likelihood ratio test for inclusion of the variable in the model.

Thus, the uPA Lia value (× 100) may be 169 ng $mg^{-1}$ protein below or 76 ng $mg^{-1}$ protein above the uPA Imubind value, which would be unacceptable for clinical purposes. This lack of agreement is by no means obvious in Figure 1. Furthermore, the standard error of $d$ is 0.027 and the 95% CI for the bias is (–0.52, –0.41). The standard error of $d \pm 2s$ is 0.047, while the 95% CI of $d - 2s$ and $d + 2s$ are (–1.78, –1.60) and (0.67, 0.85) respectively. These intervals are wide, reflecting the strong variation of the differences. They reveal discrepancies between the two methods and show that they cannot be used indifferently.

### Cut-off points for uPA Imubind and uPA Lia

In uPA Imubind, the minimum $P$-value was obtained at a cut-off of 1.84 ng $mg^{-1}$ protein when failure was taken as death, metastasis or relapse. The corrected $P$-value reached a value of < 0.0001, whatever the end point for failure, and when the range of possible cut-off points was restricted to the interval between the 5% and 95% quantiles of the uPA Imubind value. The same analysis was used to set the cut-off points for uPA Lia at 0.20 ng $mg^{-1}$ protein and 1.14 ng $mg^{-1}$ protein, with a corrected $P$-value of < 0.0001, whatever the end point for failure.

### Relation between uPA Imubind, uPA Lia and other patients characteristics (Table 1)

Whatever the technique, there was no significant link between the level of uPA and age or menopausal status. The uPA imubind value was not related to ER status, but the majority of tumours (76%) with uPA Lia < 1.14 ng $mg^{-1}$ protein were $ER^+$. Tumours with high uPA values (Imubind > 1.84 or uPA Lia > 1.14) were more often $PgR^-$. Tumours with high uPA values (Imubind > 1.84 or uPA Lia > 1.14) were more often SBR II or III or MSBR grade II. The majority of large tumours (clinical size > 25 mm, surgical size > 20 mm) contained high uPA levels by both methods. The majority (82%) of tumours larger than 25 mm contained high uPA levels by both methods. Although the frequency of patients with high uPA Imubind or uPA Lia values was approximately the same in the node-negative and node-positive subgroups, about 47% of uPA levels (uPA Imubind ≤ 1.84 or uPA Lia ≤ 0.20) were observed in node-negative patients.

### Univariate analyses (Table 2)

Age and menopausal status were not significantly related to any of the three outcomes. Overall survival was influenced by ER status, but there was no significant difference in DFS or MFS. However, all the remaining factors seemed to affect OS, DFS and MFS when examined individually. In particular, high levels of uPA Imubind (> 1.84) and uPA Lia (> 1.14) were significantly associated with poorer overall survival and shorter relapse-free or metastasis-free survival. Figure 3 displays the survival curves of uPA determined by the two techniques. There are significant differences between curves 1 (uPA Imubind ≤ 1.84) and 2 (uPA Imubind > 1.84) ($P < 0.0001$), 3 (uPA Lia ≤ 0.20) and 4 (uPA Lia [0.20–1.14]) ($P < 0.001$), and between curves 4 and 5 (uPA Lia > 1.14) ($P < 0.00001$), whatever the outcome.

### Multivariate analyses

All the variables listed in Table 1, together with adjuvant treatments (hormonal therapy and chemotherapy) were candidates in the multivariate Cox regression model for their relationships with OS, DFS and MFS.

### Overall population

The results of multivariate analyses of 499 breast cancer tumours using the Cox model are presented in Table 2. The prognostic factors independently associated with shorter overall survival were nodal status (> 3 involved nodes), uPA Imubind (> 1.84), clinical tumour size (> 25 mm) and ER negativity. Three factors were significantly related to DFS with the poorest prognosis: nodal status (> 3 involved nodes), uPA Lia (> 1.14) and clinical tumour

© Cancer Research Campaign 1998

**Table 3** Results of Cox multivariate analyses in the node-negative and node-positive subgroups of patients

| Subgroup | Outcome | Variable | *P*-value | RR[a] (CI)[b] |
|---|---|---|---|---|
| Node-negative | | | | |
| | OS | uPA Lia | 0.005 | 2.75 (1.35–5.57) |
| | DFS | uPA Lia | 0.0002 | 2.33 (1.50–3.62) |
| | MFS | uPA Lia | < 0.0001 | 3.73 (2.17–6.42) |
| Node-positive | | | | |
| | OS | Nodal status[c] | < 0.0001 | 3.21 (1.85–5.55) |
| | | uPA Imubind | < 0.0001 | 3.26 (1.86–5.72) |
| | | ER status | 0.002 | 2.25 (1.33–3.81) |
| | | Clinical size | 0.02 | 2.23 (1.04–4.78) |
| | DFS | Nodal status | < 0.0001 | 2.19 (1.45–3.31) |
| | | uPA Lia | 0.0002 | 1.70 (1.18–2.43) |
| | | MSBR grade | 0.003 | 2.21 (1.17–4.20) |
| | | Clinical size | 0.008 | 2.00 (1.15–3.46) |
| | MFS | Nodal status | < 0.0001 | 2.52 (1.59–3.98) |
| | | uPA Imubind | 0.0008 | 2.42 (1.44–4.08) |
| | | Clinical size | 0.005 | 2.17 (1.19–3.98) |
| | | ER status | 0.03 | 1.71 (1.07–2.73) |

[a]RR, relative risk. [b]CI, 95% confidence interval. [c]Nodal status is defined as 1–3 versus > 3 involved nodes in the node-positive group. For the coding of the other variables, see Table 1.

size (> 25 mm). With regard to MFS, the most important adverse prognostic factors were nodal status (> 3 involved nodes), uPA Lia (> 1.14), uPA Imubind (> 1.84), clinical tumour size (> 25 mm) and MSBR grade II.

### Nodal status subgroups

The results of Cox multivariate analyses in the node-negative and node-positive subgroups are presented in Table 3. In the subgroup of patients free from lymph node involvement, uPA Lia (> 1.14) was the only important adverse prognostic factor for OS, DFS and MFS. In the patients with node involvement, nodal status (> 3 involved nodes), uPA Imubind (> 1.84), clinical tumour size (> 25 mm) and ER negativity were significantly associated with shorter overall survival. With regard to DFS, the most important adverse prognostic factors significantly related to the risk of relapse were nodal status (> 3 involved nodes), uPA Lia (> 1.14), MSBR grade II and clinical tumour size (> 25 mm). Four factors were independently associated with shorter MFS: nodal status (> 3 involved nodes), uPA Imubind (> 1.84), clinical tumour size (> 25 mm) and ER status.

### DISCUSSION

Tumour invasion, which is associated with destruction of the basement membrane and subcellular matrix (Duffy et al, 1987), appears to be caused by the coordinated action of proteases secreted by malignant cells and the stroma. Urokinase and its inhibitors have been proposed as new prognostic factors in breast cancer (Duffy et al, 1990; Jänicke et al, 1990; Foekens et al, 1992; Spyratos et al, 1992; Bouchet et al 1994; Foekens et al, 1995). However, their use for this purpose can only be envisaged once the results obtained with different assay methods have been compared. The prognostic value of uPA assay by the Imubind method has been demonstrated (Foekens et al, 1992; Jänicke et al, 1991, 1994). The aim of our work was to test a new automated ELISA

© Cancer Research Campaign 1998

Table 4 Published distributions of uPA (ng mg⁻¹ cytosolic protein) in breast tumours

| Authors | Range | Median | Mean | Cut-off | Median follow-up (months) | Methods | Triton X-100 extract |
|---|---|---|---|---|---|---|---|
| Jänicke et al. (1990) | (0.07–11.90) | 2.60 | 3.20 | 3.49 | 25 | Imubind | Yes |
| Foekens et al. (1992) | (0.01–9.80) | 0.70 | 1.00 | 1.15 | 60 | Imubind | No |
| Jänicke et al. (1994) | (0.13–15.17) | 2.32 | 3.06 | 2.97 | 30 | Imubind | Yes |
| | (0.02–9.08) | 1.07 | 1.67 | 1.56 | 30 | Imubind | No |
| Fernö et al. (1996) | (0.00–3.19) | 0.40 | | 0.62 | 42 | Lia | No |
| Our study | (0.00–7.22) | 0.75 | 1.00 | 1.84 | 72 | Imubind | No |
| | (0.01–2.81) | 0.38 | 0.53 | (0.20, 1.14) | 72 | Lia | No |

method for uPA and to compare the results obtained with the Imubind method on the same breast cancer cytosols. The cytosols were prepared under identical conditions to those used for hormone receptor assays. The Lia uPA method is simple, rapid and highly reproducible. The between-run coefficient of variation, based on a pooled sample, was lower in the Lia than in the Imubind method (8.3% and 11.6% respectively). Table 4 compares recently published breast tumour cytosol uPA values obtained using the Imubind and Lia methods in the presence and absence of Triton X-100 in the homogenization buffer (Schmitt et al, 1991). Our Imubind results are very similar to those of Foekens et al (1992) and Jänicke et al (1994) who, like us, did not use an ionic detergent. The results obtained with the Lia uPA method on all the cytosols were similar to those reported by Fernö et al (1994, 1996) using the same method. In our study, the median value obtained in the Lia method was below that obtained in the Imubind method, confirming previous reports.

There was a good correlation between the two methods, but the correlation coefficient is not a reliable basis for demonstrating the equivalence of two methods (Bland and Altman, 1986). UPA values depend on the source and composition of the ELISA kit. Commercial antibodies have different specificities and affinities for the multiple molecular forms of urokinase, i.e. the single chain of the proenzyme (pro-uPA), the low molecular weight chain (LMW-uPA) and the high molecular weight chain (HMW-uPA). In addition, pro-uPA and HMW-uPA can be complexed to the uPA receptor (uPAR). HMW-uPA, LMW-uPA and receptor-bound uPA (uPA-uPAR) can also be complexed to the two main inhibitors of uPA (PAI-1 and PAI-2). Thus, the uPA present in cytosols occurs in a variety of structures and molecular weights. The antibodies provided with the two assay kits also have different compositions (Table 4). It is not, therefore, surprising that the observed antigen level differs when measured in the same cytosol extract, especially as the proteolytic cascade involves proenzymes and enzymes, receptors, inhibitors and antibodies with different specificities.

We used the approach of Hilsenbeck and Clark (1996) to choose the cut-offs; consequently, uPA Imubind values were dichotomized, while uPA Lia values were divided into three groups. The positions of the curves in Figure 3 justify this choice. For example, 75% of the 51 cases in group 5 (uPA Lia > 1.14) were included in group 2 (uPA Imubind > 1.84), and 22 (81%) out of the 27 patients with metastases in group 5 (uPA Lia > 1.14) were included in group 2 (uPA Imubind > 1.84). Whatever the method (Imubind or Lia) and irrespective of the cut-offs we determined, respectively 15% and 10% of patients with high uPA values had a poor prognosis. The corresponding proportions of patients were higher (26%, 32% and 33% respectively) in the studies by Jänicke et al (1990), Foekens et al (1992) and Fernö et al (1996). These differences can be explained by inequalities in the size of the populations studied and in the median follow-up periods (12.5 months, 48 months and 42 months respectively; 72 months in our study).

In the multivariate analyses, the predictive value of uPA remained high and independent of conventional predictive factors, regardless of the assay method (Imubind or Lia). However, high uPA Lia values were chiefly associated with shorter disease-free survival and metastasis-free survival in the overall population. In the subgroup of patients free of lymph node involvement, a high uPA Lia value was the only important adverse prognostic factor for OS, DFS and MFS. These results support those of Kute et al (1994) and Jänicke et al (1993).

Automated urokinase assay by the Lia method is thus feasible, and the predictive value of this marker is valid regardless of the method used. Whether one or several markers should be used to detect tumour invasion remains to be settled. Imubind ELISA methods for uPA, uPAR and the two inhibitors (PAI-1 and PAI-2) are being assessed. It is probable that two or more markers will have to be combined to obtain a reliable prognostic score in breast cancer. Assay techniques for markers of metastatic disease must be perfectly standardized (Schmitt et al, 1991; Jänicke et al, 1994; Romain et al, 1995; Benraad et al, 1996; Bouchet et al, 1996; Rønne et al, 1995) if they are to be of use for diagnosis and the decision to give adjuvant therapy.

### ACKNOWLEDGMENTS

The authors thank the Ligues Contre le Cancer, Comité des Yvelines and Comité des Hauts de Seine for their financial support.

### REFERENCES

Benraad THJ, Gerts-Moespot J, Grøndahl-Hansen J, Schmitt M, Heuvel JJTM, De Witte JH, Foekens JA, Leake RE, Brunner N and Sweep CGI (1996) Immunoassays (ELISA) of urokinase-type plasminogen activator (uPA): report of an EORTC/BIOMED-1 Workshop. *Eur J Cancer* **32A**: 1371–1381

Bland JM, Altman DG (1986) Statistical methods for assessing agreement between two methods of measurement. *Lancet* **8**: 307–310

Bloom HJG, Richardson WW (1957) Histological grading and prognosis in breast cancer. A study of 1,409 cases of which 359 have been followed for 15 years. *Br J Cancer* **11**: 359–377

© Cancer Research Campaign 1998

Bouchet C, Spyratos F, Martin PM, Hacène K, Gentile A and Oglobine J (1994) Prognostic value of urokinase-type plasminogen activator (uPA) and plasminogen activator inhibitors PAI-1 and PAI-2 in breast carcinomas. *Br J Cancer* **69**: 398–405

Bouchet C, Spyratos F, Andrieu C, Deytieux S, Becette V and Oglobine J (1996) Influence of the extraction procedure on plasminogen activator inhibitor-2 (PAI-2) and urokinase receptor (uPAR) assays in breast cancer tissues. *Breast Cancer Res Treat* **41**: 141–146

Cho JY, Chung HC, Noh SH, Roh JK, Min JS and Kim BS (1997) High level of urokinase-type plasminogen activator is a new prognostic marker in patients with gastric carcinoma. *Cancer* **79**: 878–883

Cox DR (1972) Regression models and life tables. *J R Stat Soc B* **34**: 187–220

Danø K, Andreasen PA, Grøndahl-Hansen J, Kristensen PI, Nielsen LS and Skriver L (1985) Plasminogen activators, tissue degradation and cancer. *Adv Cancer Res* **44**: 139–266

Duffy MJ (1987) Do proteases play a role in cancer invasion and metastasis? *Eur J Cancer Clin Oncol* **23**: 583–589

Duffy MJ, O'Grady P, Devaney D, O'Siorain L, Fennelly JJ and Lijnen HJ (1988) Urokinase-plasminogen activator, a marker for aggressive breast cancer. Preliminary report. *Cancer* **62**: 531–533

Duffy MJ, Reilly D, O'Sullivan C, O'Higgins N, Fennelly JJ and Andreasen P (1990) Urokinase plasminogen activator, a new and independent prognostic marker in breast cancer. *Cancer Res* **50**: 6827–6829

EORTC Breast Co-operative Group (1980) Revision of the standards for the assessment of hormone receptor in human breast cancer. Report of the second EORTC workshop, held on 16–17 March 1979, in Netherlands Cancer Institute. *Eur J Cancer* **16**: 1513–1515

Fernö M, Borg A, Killander D, Hirschberg L and Brundell J (1994) Urokinase plasminogen activator as prognostic factor in breast cancer: measured with a novel immunoluminometric assay suited for routine use. *Breast Cancer Res Treat* suppl. 32: 30

Fernö M, Bendahl PO, Borg A, Brundell J, Hirschberg L, Olosson H and Killander D (1996) Urokinase plasminogen activator, a strong independent prognostic factor in breast cancer, analysed in steroid receptor cytosols with a luminometric immunoassay. *Eur J Cancer* **32A**: 793–801

Foekens JS, Schmitt M, Pache L, Van Putten WLJ, Peters HA, Bontenbal M, Jänicke F and Klijn JGM (1992) Prognostic value of urokinase-type plasminogen activator in 671 primary breast cancer patients. *Cancer Res* **52**: 6101–6105

Foekens JS, Buessecker F, Peters HA, Krainick U, Van Putten WLJ, Look MP, Klijn JGM and Kramer MD (1995) Plasminogen activator inhibitor-2 prognostic relevance in 1012 patients with primary breast cancer. *Cancer Res* **55**: 1423–1427

Ganesh S, Sier CFM, Griffioen G, Vloedgraven H, De Boer A, Welvaart K, Van de Velde C, Van Krieken J, Verheijen J, Lamer C and Verspaget HW (1994) Prognostic relevance of plasminogen activators and their inhibitors in colorectal cancer. *Cancer Res* **54**: 4065–4071

Hasui Y, Marutsuka K, Suzumiya J, Kitada S, Osada Y and Sumiyoshi A (1992) The content of urokinase-type plasminogen activator as a prognostic factor in bladder cancer. *Int J Cancer* **50**: 871–873

Hilsenbeck SG and Clark GM (1996) Practical p-value adjustment for optimally selected cutpoints. *Stat Med* **5**: 103–112

Jänicke F, Schmitt M, Hafter R, Hollrieder A, Babic R, Ulm K, Gossner W and Graeff H (1990) Urokinase-type plasminogen activator (uPA) antigen is a predictor of early relapse in breast cancer. *Fibrinolysis* **4**: 69–78

Jänicke F, Schmitt M and Graeff H (1991) Clinical relevance of the urokinase-type and tissue-type plasminogen activators and of their type 1 inhibitor in breast cancer. *Semin Thromb Hemost* **3**: 303–312

Jänicke F, Schmitt M, Pache L, Ulm K, Harbeck N, Hofler H and Graeff H (1993) Urokinase (uPA) and its inhibitor PAI-1 are strong and independent prognostic factors in node-negative breast cancer. *Breast Cancer Res Treat* **234**: 195–208

Jänicke F, Pache L, Schmitt M, Ulm K, Thomssen C, Prechtl A and Graeff H (1994) Both the cytosols and detergent extracts of breast cancer tissues are suited to evaluate the prognostic impact of the urokinase-type plasminogen activator and its inhibitor plasminogen activator inhibitor type 1. *Cancer Res* **54**: 2527–2530

Kaplan EL and Meier P (1958) Non-parametric estimation from incomplete observation. *J Am Stat Assoc* **53**: 457–481

Kuhn W, Pache L, Schmalfeldt S, Dettmar P, Schmitt M, Jänicke F and Graeff H (1994) Urokinase (uPA) and PAI-1 predict survival in advanced ovarian cancer patients (FIGO III) after radical surgery and platinum based chemotherapy. *Gynecol Oncol* **55**: 401–409

Kute T, Gøndahl-Hansen J, Russel G, Christensen IJ and Brünner N (1994) Urokinase-type-plasminogen activator, its inhibitor and cathepsin D predicts prognosis in node negative breast cancer. *Proc Am Assoc Cancer Res* **35**: 201

Le Doussal V, Tubiana-Hulin M, Hacene K, Friedman S and Brunet M (1989) Nuclear characteristics as indicators of prognosis in node-negative breast cancer patients. *Breast Cancer Res Treat* **14**: 207–216

Nekarda H, Schmitt M, Ulm K, Wenninger A, Vogelsang H, Becker K, Roder JD, Fink U and Siewert JR (1994) Prognostic impact of urokinase-type plasminogen activator and its inhibitor PAI-1 in completely resected gastric cancer. *Cancer Res* **54**: 2900–2907

Oka T, Ishida T, Nishino T and Sugimachi K (1991) Immunohistochemical evidence of urokinase plasminogen activator in primary and metastatic tumours of pulmonary carcinoma. *Cancer Res* **51**: 3522–3525

Peto R, Pike MC and Armitage P (1977). Design and analysis of randomized clinical trials requiring prolonged observations of each patient. Part II. Analyses and examples. *Br J Cancer* **35**: 1–39

Romain S, Spyratos F, Lainé-Bidron C, Bouchet C, Guirou O, Martin PM Oglobine J and Magdelénat H (1995) Comparative study of four extraction procedures for urokinase type plasminogen activator and activator inhibitor-1 in breast cancer tissues. *Eur J Clin Chem Clin Biochem* **33**: 603–608

Rønne E, Høyer-Hansen G, Brünner N, Pedersen H, Rank F, Osborne CK, Clark GM, Danø K and Grøndahl-Hansen J (1995) Urokinase receptor in breast cancer tissue extracts. Enzyme-linked immunosorbent assay with a combination of mono- and polyclonal antibodies. *Breast Cancer Res Treat* **33**: 199–207

Schmitt M, Goretzki L, Jänicke F, Calvete J, Eulitz M, Kobayashi H, Chucholowski N and Graeff H (1991) Biological and clinical relevance of the urokinase type plasminogen activator (uPA) in breast cancer. *Biomed Biochim Acta* **50**: 731–741

Skelly MM, Mulcahy HE, Connell T, Troy A, Duggan C, Duffy MJ, Sheahan K and O'Donoghue DP (1995) Urokinase-type plasminogen activator (uPA) is independently related to outcome in colorectal cancer. *Ir J Med Sci* **164** (suppl. 14):2

Spyratos F, Martin PM, Hacène K, Romain S, Andrieu C, Ferrero-Poüs M, Deytieux S, Le Doussal V, Tubiana-Hulin M and Brunet M (1992) Multiparametric prognostic evaluation of biological factors in primary breast cancer. *J Natl Cancer Inst* **84**: 1266–1272

Wiechelman K, Braun R and Fitzpatrick J (1988) Investigation of the bicinchonic acid protein assay: identification of the groups responsible for color formation. *Anal Biochem* **193**: 265–275

© Cancer Research Campaign 1998